# Influence of Acute and Chronic Exercise on Abdominal Fat Lipolysis: An Update

**DOI:** 10.3389/fphys.2020.575363

**Published:** 2020-12-07

**Authors:** Claire Laurens, Isabelle de Glisezinski, Dominique Larrouy, Isabelle Harant, Cedric Moro

**Affiliations:** ^1^INSERM, UMR 1048, Institute of Metabolic and Cardiovascular Diseases, Obesity Research Laboratory, Toulouse, France; ^2^Paul Sabatier University, Toulouse, France; ^3^Department of Physiological Functional Explorations, Rangueil University Hospital, Toulouse, France

**Keywords:** adipose tissue, lipolysis, fatty acids, exercise, weight loss

## Abstract

Exercise is a powerful and effective preventive measure against chronic diseases by increasing energy expenditure and substrate mobilization. Long-duration acute exercise favors lipid mobilization from adipose tissue, i.e., lipolysis, as well as lipid oxidation by skeletal muscles, while chronic endurance exercise improves body composition, facilitates diet-induced weight loss and long-term weight maintenance. Several hormones and factors have been shown to stimulate lipolysis *in vitro* in isolated adipocytes. Our current knowledge supports the view that catecholamines, atrial natriuretic peptide and insulin are the main physiological stimuli of exercise-induced lipolysis in humans. Emerging evidences indicate that contracting skeletal muscle can release substances capable of remote signaling to organs during exercise. This fascinating crosstalk between skeletal muscle and adipose tissue during exercise is currently challenging our classical view of the physiological control of lipolysis, and provides a conceptual framework to better understand the pleotropic benefits of exercise at the whole-body level.

## Introduction

Physical exercise is one of the most effective lifestyle interventions to fight against many chronic diseases and in particular obesity and type 2 diabetes. Health benefits of exercise are achieved through an improvement of energy metabolism and glucose homeostasis. These benefits are sustained over the long term through an improvement of body composition induced by muscle hypertrophy and fat mass loss (Ross and Bradshaw, 2009; Peterson et al., 2011; Stoner et al., 2016; Evans et al., 2019; Hsu et al., 2019; Viana et al., 2019). Importantly, even if exercise training interventions usually have a very modest impact on body weight, a consistent observation is a reduction of waist circumference and visceral white adipose tissue (WAT) mass, and is therefore an efficient strategy to reduce cardiometabolic risk in obese individuals (Wewege et al., 2017).

During an exercise bout, skeletal muscle relies on both fatty acids (FA) and glucose as fuels to sustain muscle fiber contraction. When exercise is performed at high intensity and for a short duration, muscle cells primarily rely on glucose and muscle glycogen as fuels, which is mainly released from muscle and liver glycogen stores. However, if the exercise is performed at a moderate intensity and for a longer duration, FA will become the main source of energy to sustain muscle contraction. Indeed, FA oxidation in muscle is dependent of FA supply from different sources: FA released through lipolysis of triacylglycerols (TG) stored within WAT, from circulating very low-density lipoproteins-TG (VLDL-TG), from intramyocellular triacylglycerols (IMTG) and potentially TG stored within inter/intra-muscular adipose tissue (IMAT). The contribution of VLDL-TG to whole-body lipid oxidation varies from 5 to 10% at rest and seems to be marginal during exercise (Wolfe et al., 1985; Kiens and Lithell, 1989). Fatty acids-derived from IMTG and peripheral WAT are thus by far the major sources of lipid fuels during exercise (Horowitz, 2003). Their relative contribution to exercise energy expenditure is influenced by a number of factors such as exercise intensity, duration, and training status (Horowitz and Klein, 2000). Low to moderate intensity exercise, ranging from 25 to 65% of maximal oxygen consumption (VO_2_max), is associated with a 5 to 10-fold increase in whole-body lipid oxidation compared to rest (Romijn et al., 1993). A large part of the increase in FA availability is supplied by WAT lipolysis, which increases by 2–4 times (Romijn et al., 1993; Klein et al., 1994; Krauzova et al., 2018). In this review, we will discuss the effects of acute and chronic exercise on abdominal WAT lipolysis in lean and obese individuals.

## Effect of Acute Exercise on Adipose Tissue Lipolysis

### Main FA Sources

Numerous studies have demonstrated a tight link between lipolysis and FA oxidation during exercise. Indeed, a positive correlation has been observed between lipolytic rate measured *in vitro* in isolated adipocytes and whole-body resting FA oxidation in healthy individuals (Imbeault et al., 2000). In addition, a strong positive relationship has been described between subcutaneous abdominal WAT lipolysis and whole-body FA oxidation measured during an exercise bout in endurance-trained subjects (Moro et al., 2014). Furthermore, the adipose triglyceride lipase (ATGL) activity is increased during exercise in WAT from lean and obese individuals (Petridou et al., 2017).

The abdominal WAT is made of two main fat compartments, subcutaneous WAT (SCAT) on one side, and visceral WAT on the other side. Exercise mostly activates lipolysis in SCAT, as only 5–10% of circulating long-chain FA are released from visceral adipose tissue in lean subjects (Horowitz, 2003; Nielsen et al., 2004). Abdominal SCAT lipolytic response is dependent on both exercise intensity and duration (Horowitz, 2003). In addition, it has been suggested that subcutaneous abdominal lipolysis is greater than gluteo-femoral lipolysis both in men and women, and that men have a relative “resistance” to norepinephrine-mediated lipolysis due to higher adipocyte content of alpha2-adrenergic receptors that inhibits lipolysis (Leibel and Hirsch, 1987; Jensen and Johnson, 1996; Moro et al., 2007). However, in these studies, the relative lipolytic rate measured *in vitro* on isolated adipocytes, *in situ* through microdialysis, and *in vivo* using A–V differences appeared similar between men and women, thus indicating that the greater lipid mobilization observed during exercise in women is mostly accounted for by a higher subcutaneous fat mass compared to men.

### Main Lipolytic Hormones and Factors

The activation of SCAT lipolysis during exercise can be attributed to an increase in plasma catecholamines concentration, which stimulates beta-adrenergic receptors on the adipocyte plasma membrane leading to the intracellular activation of the hormone-sensitive lipase (HSL; Horowitz, 2003). However, we have previously shown that local infusion of the beta-blocking agent propranolol in the SCAT only partially inhibits exercise-induced lipolysis (Moro et al., 2004; Verboven et al., 2018). The 60–70% residual lipolysis was found to correlate with plasma atrial natriuretic peptide (ANP) concentration (Moro et al., 2004, 2008). The role of ANP in exercise-induced lipolysis was then further confirmed during repeated bouts of endurance exercise in lean healthy and obese individuals (Moro et al., 2006; Koppo et al., 2010). Thus, besides the well-known role of catecholamines on exercise-induced WAT lipolysis, the increase of plasma ANP, along with the decrease of plasma insulin (Moro et al., 2007), in relation to exercise intensity actively contribute to enhance adipocyte lipolysis during exercise. Interestingly, when exercise is performed the day after an exercise-bout when muscle glycogen stores are still low, lipolysis is increased compared with the same exercise performed after a resting day, in elite cyclists (Moro et al., 2014). Strikingly, this observation could not be explained by the aforementioned classical lipolytic agents, therefore suggesting that other factors may participate in the activation of WAT lipolysis during exercise (Moro et al., 2014). Recent evidence indicate that proteins secreted by muscle fibers during contraction, the so-called myokines, could activate WAT lipolysis in humans. Indeed, interleukin-6 (IL-6) was the first myokine to be discovered and IL-6 plasma levels were increased in response to an acute exercise bout (Pedersen et al., 2001; Reihmane and Dela, 2014). A recent clinical study has demonstrated that IL-6 is required to reduce visceral adipose tissue mass in response to exercise training (Wedell-Neergaard et al., 2019). However, the role of IL-6 in the activation of WAT lipolysis is still a matter of debate, as IL-6 acute treatment does not activate adipocyte lipolysis *in vitro* (Trujillo et al., 2004). In addition, an acute elevation of IL-6 *in vivo* was described to increase whole-body lipolysis due to a rise in muscle FA release, while WAT lipolysis remained unchanged (Wolsk et al., 2010). Irisin is another myokine that has been described to increase WAT lipolysis through an indirect mechanism involving WAT browning (Bostrom et al., 2012). However, altthough some experiments performed in rodents suggest that exercise-released myokines may activate WAT browning (Stanford et al., 2015), the relevance of such mechanism in humans remains controversial (Norheim et al., 2014; Lehnig and Stanford, 2018).

More recently, we identified a novel myokine secreted by contracting human primary skeletal muscle cells, called growth and differentiation factor 15 (GDF15), which enhances adipocyte lipolysis *in vitro* (Laurens et al., 2020). Furthermore, GDF15 was also secreted following both high-intensity or moderate-intensity exercise in humans *in vivo*, and recombinant GDF15 protein was able to activate lipolysis in subcutaneous WAT explants (Laurens et al., 2020).

White adipose tissue has also been described to produce soluble factors that may act in a paracrine/autocrine fashion to sustain lipolysis during exercise such as interleukin-15 (IL-15). It has been demonstrated that IL-15 can be produced by SCAT during a one h-cycling exercise, which is known to increase WAT lipolysis. In addition, resting IL-15 secretion correlates with SCAT lipolysis, and an infusion of IL-15 through microdialysis activates SCAT lipolysis in lean subjects while it suppresses lipolysis in obese subjects (Pierce et al., 2015). However, no correlations were observed between IL-15 secretion and lipolysis during exercise. Thus, whether IL-15 contributes to exercise-induced lipolysis is still debated and warrants further investigations.

### Exercise Intensity

The relative contribution of FA utilization during an exercise bout depends on its intensity. White adipose tissue lipolysis increases from low to moderate intensities and decreases at high intensity (Romijn et al., 1993). Indeed, when exercise is performed at high intensity, glucose is the major energy substrate to rapidly fuel the contracting muscle. However, as exercise intensity decreases, a switch occurs and lipids become the major energy substrate (i.e., crossover concept) (Brooks and Mercier, 1994). The concept of “Fatmax” has then been taken up by Jeukendreup and colleagues to describe the exercise intensity, expressed as a percent of VO_2_max, eliciting the maximal reliance on fat as fuel oxidized in skeletal muscle (Jeukendrup and Wallis, 2005). At this intensity, half of the FA oxidized by muscle fibers are supplied by WAT lipolysis, the remaining part being intracellularly provided by IMTG pools. Fatmax value differs for each individual and mostly depends on body weight, diet, sex, and training status (Jeukendrup and Wallis, 2005). For instance, the Fatmax has been measured at 48% of VO_2_max in a large cohort of lean sedentary individuals, while it was around 65% in endurance trained subjects (Achten et al., 2002; Jeukendrup and Wallis, 2005). Interestingly, Fatmax was found to be lower in men than in women (45% vs 52% of VO_2_max, respectively) (Jeukendrup and Wallis, 2005). As stated earlier, the greater lipid oxidation at a given exercise intensity in women is accounted for by a higher lipid mobilization at a same relative exercise intensity due to a higher subcutaneous fat mass. Furthermore, Fatmax is lower in obese than in lean individuals (Perez-Martin et al., 2001). However, even if Fatmax has been widely used in exercise-based weight-loss programs, this concept has also raised some criticisms. First, Fatmax is highly dependent on diet and nutritional state, as the body relies more on carbohydrates (CHO) as fuel when they are highly available such as in postprandial conditions. Second, FA oxidation rate is similar in a large range of exercise intensities, usually from about 45 to 75% of maximal aerobic capacity, and thus does not differ much from the peak value (i.e., Fatmax value). Third, the amount of FA burned throughout 24 h not only depends on FA oxidized during exercise but also during the post-exercise recovery period, especially when exercise is performed at high intensity. Finally, Fatmax is a rate of FA oxidation, but the total amount of FA utilized is dependent on energy expenditure and high intensity exercise elicits the largest energy expenditure. Thus, training at Fatmax intensity may not confer further weight-loss benefit than other training interventions performed at higher exercise intensities.

### Exercise Duration

The contribution of FA to fuel the contracting muscle also depends on exercise duration. Studies from different groups have shown that FA oxidation gradually increases during a prolonged exercise bout while CHO oxidation decreases (Ravussin et al., 1986; Klein et al., 1994). This goes along with an increase of lipolysis with exercise duration (de Glisezinski et al., 2003; Lafontan et al., 2008). Interestingly, it has been shown that the activity of muscle HSL decreases during a prolonged exercise bout (Watt et al., 2003). This is a consequence of the increased uptake of circulating FA by muscle fibers, which in turn decreases lipolysis and oxidation of IMTG stores. The increase of WAT lipolysis is mostly due to the increase of plasma levels of pro-lipolytic hormones during prolonged exercise. Indeed, catecholamines secretion increases as a function of exercise duration. This increase is more pronounced for epinephrine than for norepinephrine, probably due to a slightly lower glycemia (de Glisezinski et al., 2003) and to the fact that norepinephrine secretion is mostly impacted by exercise intensity (Leuenberger et al., 1993). In line with this observation, we have previously demonstrated that epinephrine is the main beta-adrenergic agent contributing to exercise-induced lipolysis in SCAT (de Glisezinski et al., 2009). We have shown that this increase of adipocyte lipolysis is not only dependent on the beta-adrenergic stimulation by catecholamines but also on the reduction of plasma insulin level and the increase of plasma ANP (Arner et al., 1990; Moro et al., 2004). For instance, ANP plasma level was found to be particularly high after running a marathon, and could participate in the activation of WAT lipolysis to compensate the acute elevation of energy demand during long-distance running (Niessner et al., 2003).

Finally, the whole energy expenditure elicited by exercise has also to be taken into account when considering the contribution of FA burned in response to exercise, as a high percentage does not always reflect a large amount of FA burned if the energy expenditure elicited by the exercise bout is low. Exercise energy expenditure is linked to both exercise intensity and duration.

### Impact of Osbesity

Importantly, we and others have observed that exercise-induced SCAT lipolysis is lower in obese subjects than in non-obese subjects (Stich et al., 2000; Mittendorfer et al., 2004; Ross and Bradshaw, 2009). This was attributed to a higher sensitivity of anti-lipolytic alpha2-adrenergic receptors and a lower sensitivity of pro-lipolytic beta-adrenergic receptors in obese subjects (Stich et al., 2000). However, due to the higher fat mass in obese versus non-obese individuals, plasma FA concentration was higher in obese individuals both at rest and during exercise (Stich et al., 2000). In addition, the expression of the ANP clearance receptor NPRC is higher in adipocytes from obese subjects than in lean healthy individuals, and could participate in a lower lipolysis activation in response to ANP secretion during exercise (Dessi-Fulgheri et al., 2003; Kovacova et al., 2016; Ryden et al., 2016). Thus, while basal lipolytic rate is higher in obese vs non-obese subjects, exercise-induced lipolysis is reduced in obese subjects. This adaptive response in obesity could be seen as a protective mechanism to avoid excessive release of FA into the bloodstream during an exercise bout.

## Increased Lipolysis During Post-Exercise Recovery

The relationship between exercise intensity and FA oxidation, and therefore FA release from WAT lipolysis, is not as straightforward as initially thought. The role of FA as nutrients during post-exercise recovery has been described in a recent review by Bente Kiens’ group (Lundsgaard et al., 2020). Briefly, even if high-intensity exercise (i.e., performed at an intensity over 75% of the subject’s maximal aerobic power) elicits a low FA oxidation rate during the exercise bout, the post-exercise FA utilization is higher than after a low intensity exercise bout (Pillard et al., 2010). This greater FA oxidation after a high-intensity exercise bout is mainly reflected by a decrease of the respiratory quotient (Marion-Latard et al., 2003) and appears independent of energy expenditure during 6 h post-exercise. This is a consequence of the preferential use of CHO to replenish muscle glycogen stores which have been depleted during the high-intensity exercise bout, which favors FA as major fuels during 24–48 h after the exercise bout (Tremblay et al., 1994; Kiens and Richter, 1998). We have previously shown in isolated adipocytes that, after a long-duration exercise bout, the WAT displays an increased responsiveness to beta-adrenergic lipolytic agents, which may participate to the increased FA availability during the recovery period (Harant et al., 2002). Strikingly, this increase in post-exercise FA consumption is more pronounced in men than in women (Henderson et al., 2007). In addition, using stable isotope-labeled palmitate infusion, Magkos et al. (2009) have observed that the exercise-induced increase of FA utilization during the post-exercise recovery period is greater in subjects with a low resting plasma FA availability and is greater after an exercise resulting in high energy demand. Interestingly, it has been demonstrated that post-exercise lipolysis is stimulated in the SCAT by an increase of plasma growth hormone level, which is secreted by somatotropic cells during the exercise bout (Enevoldsen et al., 2007). A recent study performed in mice also evoked a role of IL-6, a myokine secreted by skeletal muscle fibers during exercise, in the regulation of WAT lipid metabolism during the exercise recovery (Knudsen et al., 2017).

In summary, it appears critical to consider the post-exercise recovery period to fully assess the impact of different exercise modalities on FA utilization and thus body weight loss.

## Effect of Exercise Training on Adipose Tissue Lipolysis

Exercise training improves FA mobilization during an exercise bout. Indeed, it has been shown that FA appearance rate (Ra) in the blood is higher in endurance trained subjects compared to sedentary controls (Coggan et al., 2000). Exercise training affects both the sensitivity of WAT to catecholamines, but also their secretion during exercise, which is reduced in response to a given absolute workload after training (Kjaer et al., 1987; Riviere et al., 1989; Arner, 1995). Transversal studies performed on SCAT adipocytes have suggested that beta-adrenergic sensitivity is higher in trained subjects than in sedentary controls (Crampes et al., 1986; Crampes et al., 1989; Riviere et al., 1989). In addition, longitudinal studies have demonstrated that endurance training improves the beta-adrenergic lipolytic response of isolated adipocytes in obese subjects (De Glisezinski et al., 1998a; Moro et al., 2009).

Furthermore, exercise training improves ANP responsiveness in obese subjects, but it is yet unclear whether this is due to an increase of ANP plasma concentration or to an increase of ANP receptors on the adipocyte cell surface (Moro et al., 2005). Indeed, we were able to show through *in situ* microdialysis experiments in SCAT of young overweight men, that 4 months of aerobic training improve both beta-adrenergic and ANP lipolytic responses (Stich et al., 1999; Moro et al., 2005). Finally, insulin concentration decreases with training status but the impact on WAT lipolysis is partly counterbalanced by an improvement of WAT insulin sensitivity with exercise training (Polak et al., 2005; Riis et al., 2019). Strikingly, even if exercise-induced lipolysis is higher in trained subjects, plasma FA concentration is lower both at rest and during exercise (Crampes et al., 2003; de Glisezinski et al., 2003). This could be explained by an increase of FA utilization by skeletal muscle in trained subjects. Indeed, the amount of both resting and exercise-induced FA oxidation is higher after a training program, resulting in an increased oxygen consumption (de Glisezinski et al., 2003). The improvement of exercise-induced lipolysis observed in endurance-trained obese subjects also seems to be partially due to a reduction of the anti-lipolytic effect of alpha2-adrenergic receptors in the SCAT, which may be a consequence of a lower epinephrine plasma levels, the main alpha2-adrenergic ligand. Indeed, the anti-lipolytic activity of alpha2-adrenergic receptors was reduced after endurance training in lean and obese subjects (De Glisezinski et al., 2001; Richterova et al., 2004). Interestingly, similar adaptations of WAT lipolytic response have been found after a resistance training program in obese individuals (Polak et al., 2005).

Finally, it has been observed that the exercise intensity which elicits the higher lipolytic rate is increased with exercise training (Perez-Martin et al., 2001; Achten et al., 2002). Thus, while the maximal FA utilization is reached at intensities of 30% of maximal aerobic power in sedentary subjects, it is achieved around 65% in trained individuals. This means that the total amount of FA mobilized during an exercise bout is higher in trained subjects because both energy expenditure and the percentage of FA used are increased. In addition, high intensity training elicits a gain of muscle mass which impacts basal metabolic rate and thus may increase energy expenditure and consequently impact FA oxidation during resting periods and body weight loss (Heydari et al., 2012; Osawa et al., 2014; Schubert et al., 2017; Batrakoulis et al., 2018).

Altogether, these data suggest that an exercise training program combining high-intensity and moderate intensity exercise bouts could optimize daily FA utilization and optimize body weight loss in overweight or obese individuals.

## Impact of Diet and Time of the Day on Exercise-Induced Lipolysis

Carbohydrates availability influences exercise-induced lipolysis. Indeed, glucose ingestion during an exercise bout reduces SCAT lipolysis and partially inhibits FA oxidation (De Glisezinski et al., 1998b). Exercising in the fasting state has been shown to increase FA oxidation and whole-body lipolysis in healthy subjects (Vicente-Salar et al., 2015; Andersson Hall et al., 2016; Hansen et al., 2017). This appears to be a compelling approach to achieve maximal fat utilization during exercise. Interestingly, a recent study has shown that exercising after a high-protein breakfast has similar effects on lipolysis than exercising in the fasting state (Saghebjoo et al., 2020). Furthermore, volunteers fed for 5 days with a high-fat diet display a higher WAT lipolytic rate during exercise than people fed with a CHO-rich diet, which can be explained by a higher catecholamine response and lower insulinemia (Suljkovicova et al., 2002).

Numerous review articles have described the impact of time of the day on exercise efficiency, but very few focused on lipid metabolism and WAT lipolysis (Chtourou and Souissi, 2012; Seo et al., 2013; Dollet and Zierath, 2019; Parr et al., 2020). A few studies have shown that exercise performed during the evening elicits a higher reliance on lipids compared to exercise performed during the morning (Aoyama and Shibata, 2020). In addition, a crossover study performed in young men has demonstrated that an endurance exercise bout performed during the evening enhances plasma epinephrine, IL-6 and plasma FA levels compared to the same exercise performed during the morning, thus suggesting that evening exercise is the most effective to achieve high rates of WAT lipolysis (Kim et al., 2015). However, data are still scarce and future studies should be performed to fully address this question.

## Calorie Restriction and Exercise Induced Weight Loss

Many studies have shown that calorie restriction is more efficient at reducing body weight than exercise training, and that combining exercise training with a caloric restriction intervention confers a slight additional benefit to achieve weight loss compared to calorie restriction alone (Miller et al., 1997; Swift et al., 2018). However, exercise has an important role in body weight maintenance after weight loss (Swift et al., 2018). Indeed, calorie restriction-induced weight loss increases WAT sensitivity to lipolytic stimuli produced during exercise (Mauriege et al., 1999). Furthermore, exercise protects against loss of lean body mass during calorie restriction, and avoids a drop of resting metabolic rate (Chomentowski et al., 2009).

Therefore, even if combining exercise to a calorie restriction intervention does not achieve further weight loss than calorie restriction alone, exercise potentiates visceral fat mass loss and a sustained improvement of body composition (You et al., 2006), and prevents from the well-described “yo-yo” effect of dieting.

## Current Gaps in Research

There are many additional questions that remains to be answered to fully understand the impact of exercise on WAT lipolysis and body composition. Indeed, future studies should aim at identifying unknown lipolytic factors secreted during exercise, such as myokines and potentially micro-RNAs released in extracellular vesicles in response to muscle contraction (Whitham et al., 2018). Understanding the complex inter-organ crosstalk during exercise will pave the way to new areas of research and could lead to the discovery of new molecular players with a potential therapeutic role.

Finally, research efforts should also focus on refining exercise training modalities to achieve a maximal and sustained improvement in body composition, especially in overweight or obese individuals. Assessing the combination of time-restricted eating patterns with exercise training sessions performed during the fasting state could be an attractive approach to potentiate fat mass loss.

## Conclusion

Collectively, there is little debate that exercise training facilitates abdominal weight loss in overweight and obese individuals. Chronic exercise has largely demonstrated its ability to facilitate weight loss during calorie restriction and maintenance of long-term weight loss. A number of studies suggest that combining moderate and high intensity exercise can provide additional benefits on weight loss, at least in part, by favoring higher rates of energy expenditure during exercise and greater FA oxidation rates during post-exercise recovery. Although canonical lipolytic systems and hormones have been studied in detail during the past 30 years, more recent studies uncovered a muscle-adipose tissue crosstalk mediated by myokines regulating WAT lipolysis. However, much remains to be discovered. With the discovery that contracting muscles can produce myokines capable of remotely targeting organs, including WAT, our current knowledge will likely be challenged in the next few years.

## Author Contributions

CL and CM wrote and revised the manuscript. IG, IH, and DL edited and revised the manuscript. All authors contributed to the article and approved the submitted version.

## Conflict of Interest

The authors declare that the research was conducted in the absence of any commercial or financial relationships that could be construed as a potential conflict of interest.
